# Oral histidine affects gut microbiota and MAIT cells improving glycemic control in type 2 diabetes patients

**DOI:** 10.1080/19490976.2024.2370616

**Published:** 2024-07-03

**Authors:** Moritz V. Warmbrunn, Ilias Attaye, Judith Aron-Wisnewsky, Elena Rampanelli, Eduard W.J. van der Vossen, Youling Hao, Annefleur Koopen, Per-Olof Bergh, Daniela Stols-Gonçalves, Nadia Mohamed, Marleen Kemper, Xanthe Verdoes, Koen Wortelboer, Mark Davids, Eugeni Belda, Sébastien André, Stanley Hazen, Karine Clement, Bert Groen, Daniel H. van Raalte, Hilde Herrema, Fredrik Backhed, Max Nieuwdorp

**Affiliations:** a Department of Internal and Vascular Medicine, Amsterdam University Medical Centers, Amsterdam, The Netherlands; b Amsterdam Gastroenterology and Hepatology, Amsterdam Gastroenterology Endocrinology Metabolism (AGEM) Research Institute, Amsterdam, The Netherlands; c Amsterdam Cardiovascular Science research institute, Amsterdam, The Netherlands; d Assistante Publique Hôpitaux de Paris, Nutrition Department, Pitié-Salpêtrière Hospital, CRNH Ile de France, Paris, France; e INSERM, Nutrition and Obesities, Systemic Approaches (NutriOmics), Sorbonne Université, Paris, France; f Amsterdam Amsterdam institute for Infection and Immunity (AII), Amsterdam, The Netherlands; g Department of Molecular and Clinical Medicine/Wallenberg Laboratory, Institute of Medicine, University of Gothenburg and Sahlgrenska, Gothenburg, Sweden; h Department of Cancer Biology, Lerner Research Institute, Cleveland Clinic, Cleveland, OH, USA; i Diabetes Center, Department of Internal Medicine, Amsterdam University Medical Centers, Amsterdam, The Netherlands

**Keywords:** Microbiome, histidine, insulin resistance, monocytes, diabetes

## Abstract

Amino acids, metabolized by host cells as well as commensal gut bacteria, have signaling effects on host metabolism. Oral supplementation of the essential amino acid histidine has been shown to exert metabolic benefits. To investigate whether dietary histidine aids glycemic control, we performed a case-controlled parallel clinical intervention study in participants with type 2 diabetes (T2D) and healthy controls. Participants received oral histidine for seven weeks. After 2 weeks of histidine supplementation, the microbiome was depleted by antibiotics to determine the microbial contribution to histidine metabolism. We assessed glycemic control, immunophenotyping of peripheral blood mononucelar cells (PBMC), DNA methylation of PBMCs and fecal gut microbiota composition. Histidine improves several markers of glycemic control, including postprandial glucose levels with a concordant increase in the proportion of MAIT cells after two weeks of histidine supplementation. The increase in MAIT cells was associated with changes in gut microbial pathways such as riboflavin biosynthesis and epigenetic changes in the amino acid transporter SLC7A5. Associations between the microbiome and MAIT cells were replicated in the MetaCardis cohort. We propose a conceptual framework for how oral histidine may affect MAIT cells via altered gut microbiota composition and SLC7A5 expression in MAIT cells directly and thereby influencing glycemic control. Future studies should focus on the role of flavin biosynthesis intermediates and SLC7A5 modulation in MAIT cells to modulate glycemic control.

## Introduction

Recent projections estimate that globally 1 in 8 adults will be living with diabetes in 2045.^1^ The first line of glycemic control in type 2 diabetes (T2D) is stimulation of physical activity and weight management to obtain and maintain >5% weight-loss.^2^ High protein and low carbohydrate diets can be an essential start of weight management in T2D^3^ and can contribute to improved glycemic control. Upon ingestion, dietary proteins are metabolized by gastric acid and pepsin, and further hydrolyzed by pancreatic and intestinal brush-border enzymes in the small intestines.^4,5^ Absorbed amino acids promote anabolic processes such as muscle synthesis^6^ whereas unabsorbed or undigested proteins and amino acids are fermented in the large intestine by gut microbiota.^7^ Microbially produced metabolites from amino acids can have beneficial or detrimental effects on metabolism.^8^ For example, short-chain fatty acids produced by fermented fibers are associated with improved insulin sensitivity,^9^ whereas p-cresol sulfate^10^ and trimethylamine N-oxide (TMAO) are associated with cardiovascular diseases.^11^

In this regard, diet-induced obese rats supplemented with histidine supplementation improve markers of inflammation such as TNF-α, CRP and IL-6 in adipose tissue.^12^ In humans, dietary histidine intake is inversely associated with body mass index (BMI) and fasting blood glucose.^13^ Furthermore, serum histidine concentrations are inversely associated with markers of inflammation and oxidative stress as well as reduced mortality in chronic kidney disease patients,^14^ women with obesity^15^ and patients with chronic obstructive pulmonary disease (COPD).^16^ In line, supplementation of the essential amino acid histidine in women with metabolic syndrome has been shown to improve insulin resistance, markers of inflammation and oxidative stress.^17^ However, there are contradicting results in the literature, which might be driven by different histidine derived metabolites that can exert differential effects. In this regard, the histidine microbiota-catabolism-derived metabolite Imidazole propionate (ImP) was shown to impair metabolic signaling pathways *in vitro* and in mice.^18,19^

While monocytes and macrophages are classically viewed as the key inflammatory players in insulin resistance and T2D, the role of T cells in diabetes is gaining attention. Particularly, the innate-like mucosa associated invariant T (MAIT) cells which numbers are decreased in patients with T2D and MAIT cell abundance is associated to increased HbA1c and inflammatory markers.^20^ In fact, circulating MAIT cells are not only lower in individuals with T2D but also in individuals with metabolic syndrome.^21,22^ MAIT cells are a subset of T cells that play an important role in immune defense against viral and bacterial infections and are abundant in the gut, lung, skin and circulation.^23^ In peripheral blood, MAIT cells comprise 2% to 10% of the total T cell population.^24^ Previous reports comparing germ free and conventional mice demonstrated that the gut microbiota are necessary for the presence of MAIT cells.^25^ MAIT cells express a semi-invariant T cell receptor (TCR), restricted with an invariant TCR α-chain (Vα7.2- Jα33 in humans) and limited number of β-chains^26^; their TCR is restricted to the MR1 non-classical MHC class I molecule presenting microbiome-derived metabolites from folic acid (vitamin B9) and riboflavin (vitamin B2) biosynthesis to activate MAIT cells and signal microbial infection.^27,28^ However, the exact mechanism how MAIT cells might affect glycemic control and how they could be related to gut microbiota dysbiosis is still unknown.

To better understand how the essential amino acid histidine affects gut microbiota and glycemic control, we performed a case–control clinical intervention study in patients with type 2 diabetes and healthy controls. We present a conceptual framework on how oral amino acids can modulate gut microbiota and MAIT cells to improve glycemic control.

## Methods

### Study design

**Figure 1.**
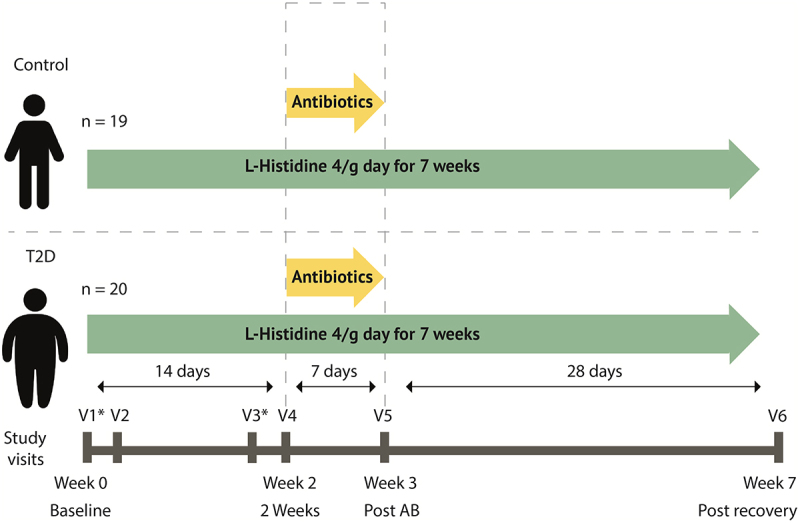
Study design. Participants use 4g of histidine daily. At day 14 antibiotics are added to the regimen for 7 days containing ciprofloxacin 500 mg once/day, metronidazole 500 mg twice/day, oral vancomycin 500 mg four times/day. At visit 2, 4, 5 and 6 feces was collected, blood was drawn and anthropometric measurements were performed. V1: visit 1. *mixed meal test was performed. Continuous glucose measure devices were used during the first four weeks and the last two weeks.

We performed a seven-week case–control clinical trial with two groups; a group of individuals with type 2 diabetes and a control group. Participants came to our study facility for were six study visits, visit 1 and 2 in week 0, visit 3 and 4 in week 2, visit 5 in week 3 and visit 6 in week 7 (Figure 1). During Visits 1 and 3, mixed meal test were performed and at visit 2, 4, 5, and 6 feces and blood samples were collected, the latter during 6 h. After baseline visit 2, participants started to consume 4 g of L-histidine per day administered in four capsules (Vital Cell Life L-Histidine 500 MG Capsules 100CP, Bunnik, The Netherlands) until the end of the study (i.e. visit 6). Oral histidine supplementation has been found safe and without side effects in a previous study during 12 weeks daily intake.^17^ To suppress their intestinal microbiota, after visit four participants used antibiotics (ciprofloxacin 500 mg once/day, metronidazole 500 mg twice/day, oral vancomycin 500 mg four times/day) for 7 days together with the oral histidine after which visit 5 was planned. After visit 5, a recovery period of 4 weeks was planned until the final visit 6. Participants were overnight fasted before all visits and all study visits took place at the Amsterdam University Medical Centers, location Academic Medical Center. The study was approved by the Ethical review Board of Academic Medical Centers of the University of Amsterdam and registered in the Dutch trial registry (https://onderzoekmetmensen.nl/nl/trial/20133) registration number: NL8372, registration date 11 February 2020. All participants provided written informed consent, and the study was performed in accordance with the Declaration of Helsinki (updated version 2013). Based on the power analysis, 40 subjects were estimated necessary (See supplemental methods).

### Study recruitment

Participants were recruited via advertisements in newspapers and online advertisements. Inclusion criteria for all participants was age of 40–70 years and Caucasian or South-Asian descent. For the control group, a BMI ranging from 19 to 25 kg/m2 was used. Participants with T2D had a BMI between 25 and 35 kg/m^2^, used metformin, a statin and had to be on stable (anti-diabetic) drug treatment for at least three months. Exclusion criteria for all groups included, GLP-1 or insulin use, proton pump inhibitor use, antibiotics use in the past three months, previous major cardiovascular events, pregnancy, chronic illnesses (history of heart failure, eGFR <30 ml/min, pulmonary disease, gastrointestinal disorders or hematologic diseases) or other inflammatory diseases. CONSORT flow chart is presented in supplemental methods Figure S1.

### Histidine measurement

Targeted measurement of histidine kinetics during 6 h was performed as previously published,^19^ with small adaptations. Briefly, 25 µL of blood plasma samples were precipitated and diluted in glass vials using acetonitrile containing internal standards (L-histidine-D_5_^15^N_3_, Cambridge Isotope Lab. Inc, imidazole propionate-13C3 and urocanate-13C3, Astra Zeneca, Cambridge, UK). After vortexing and centrifugation, the supernatant was transferred to new glass vials and evaporated under a stream of nitrogen. The samples were then reconstituted in 5% HCl (37%) in 1-butanol and placed in an oven at 70°C for 1 h allowing the n-butyl ester to be formed. After derivatization, the samples were evaporated and reconstituted in 150 µL of water:acetonitrile [90:10]. The samples were then analyzed using ultra-performance liquid chromatography coupled to tandem mass spectrometry (UPLC-MS/MS). The analytical system consisted of an Acquity UPLC I-class binary pump, sample manager and column manager coupled to a Xevo TQ-XS (Waters, Milford, MA, USA). The samples (2 µl) were injected onto a C18 BEH column (2.1×50 mm with 1,7 µm particles, Waters, Milford, MA, USA) and separated using a gradient consisting of water with 0.1% formic acid (A-phase) and acetonitrile with 0.1% formic acid (B-phase). The analytes were detected with multiple reaction monitoring using the transitions 212/110 (histidine), 197/81 (imidazole propionate) and 195/93 (urocanate). For the internal standards, the transitions 220/118, 200/82 and 198/95 respectively were used. Calibration curves of histidine, imidazole propionate and urocanate were made in methanol and treated the same way as the samples.

### Mixed meal test

At visit 1 and visit 3, participants underwent a 2 h mixed meal test to assess glycemic control, insulin sensitivity and insulin secretion in a more physiologic setting compared to an oral-glucose tolerance test.^29,30^ In short, participants were fasted for at least 8 h before the visit and an intravenous catheter was positioned in a distal arm vein. After baseline sample collection, participants ingested a liquid meal (Nutridrink, Nutricia Advanced Medical Nutrition, Amsterdam, the Netherlands), which contained 500 kcal in 250 ml (49% carbohydrates, 35% fat, 16% protein). Blood was drawn before ingestion and after 30, 60, and 120 min, centrifuged and stored at −80°C for further analysis. Glucose and insulin (Atellica IM, Siemens, The Hague, the Netherlands) levels were measured at the clinical laboratory of the Amsterdam University Medical Centers, location Amsterdam Medical Centers, conform to local protocol.^31^

### PBMC isolation and flow cytometry analysis

After blood collection in EDTA tubes, samples were stored for 4 h at 4°C. PBMC were isolated by using Lymphoprep (GE Healthcare). PBMCs were stored in cryotubes with 10% DMSO in fetal bovine serum (FBS) and immediately cooled down to −80°C in a freezing container Mr. Frosty (Thermo scientific). After 24 h, the samples were stored in liquid nitrogen. After the completion of the trial, PBMC of all participants, isolated at baseline and after 2 weeks of histidine supplementation, were thawed in 10 ml of 10%FBS-RPMI 1640 medium. DMSO was removed by centrifugation at 350 g for 10 min at 4°C. One million PBMCs were stained for immunophenotyping different immune cell subsets. Prior to antibody staining, cells were incubated for 10 min with Human TruStain FcX Fc Receptor Blocking solution (BioLegend). PBMC were stained in 200ul 1%BSA in 2 mM EDTA-PBS for 20 min at 4°C with the following antibodies: Brilliant Violet 421™ anti-human CD192 (CCR2) Antibody, Brilliant Violet 650™ anti-human CD86 Antibody, Brilliant Violet 605™ anti-human CD163 Antibody, Brilliant Violet 421™ anti-human CD206 (MMR) Antibody, Alexa Fluor® 488 anti-human CD11b Antibody, APC anti-human CD14 Antibody, PerCP/Cyanine5.5 anti-human CD16 Antibody, Brilliant Violet 510™ anti-human CX3CR1 Antibody, PE anti-human CD45 Antibody, Brilliant Violet 605™ anti-human CD161 Antibody, Brilliant Violet 421™ anti-human CD127 (IL-7 Rα) Antibody, Brilliant Violet 711™ anti-human TCR Vα7.2 Antibody, Brilliant Violet 421™ anti-human CD69 Antibody (from Biolegend), Alexa Fluor 488 anti-CD3 Monoclonal Antibody (OKT3), Alexa Fluor 700 anti-CD4 Monoclonal Antibody (RPA-T4), eFluor 506 anti-CD8a Monoclonal Antibody (RPA-T8), Super Bright 600 anti-CD25 Monoclonal Antibody (BC96), Super Bright 702 anti-CD38 Monoclonal Antibody (HIT2) (from eBioscience™). Afterwards, cells were washed three times in 1%BSA EDTA-PBS and fixed for 15 min at room temperature in 2% paraformaldehyde-PBS. Stained cells were analyzed on a BD LSRFortessa™ Cell Analyzer and data analysis was performed with the FlowJo 10.1r5 software (Tree Star). MAIT cells were identified by the expression of CD161 and the invariant TCR Vα7.2 chain. For gating strategy see Figures S8-S9.

Procedures for flow cytometry of MAIT cells in the MetaCardis replication cohort were previously published.^20^

### Continuous glucose measurement

To more accurately profile 24-h differences in glucose control between visits, the CGM was worn the first 4 weeks and 2 weeks before visit 6, used a continuous glucose monitor (FreeStyle Libre 1 sensor and scanner, Abbott) for 2 weeks. Participants were asked to scan at least every 8 h and refrain from any dietary or behavioral adaptations changes during the study. Data were processed and derivatives were calculated with the CGDA library^32^ in R (v 4.2.1).

### Fecal sample preparation and microbiota analyses

Before visits 1, 3, 5 and 6, participants collected fresh 24 h fecal samples and participants were asked to freeze the samples at −20°C and transport samples in a cooling bag. Once at the research facility, samples were immediately frozen at −80°C. Deoxyribonucleic acid (DNA) extraction and library composition were performed as previously published.^33,34^ In short, fecal samples were lysed in Lysing Matrix E tubes (MP Biomedicals) containing ASL buffer (Qiagen). Cells were lysed after homogenization by 2 min vortexing with subsequent two heating cycles at 90°C for 10 min followed by three bead beating burst at 5.5 m s^−1^ for 60 s in a FastPrep-24 instrument (MP Biomedicals). Samples were cooled on ice after each bead-beating burst for 5 min. The fecal DNA supernatants were collected after two centrifugation cycles at 4°C. Supernatants of the centrifugation steps were pooled, and aliquots of 600 µL from each sample was purified with the QIAamp DNA Mini kit (QIAGEN) in the QIAcube instrument (QIAGEN) by the human DNA analysis procedure. Using 200 µL of AE buffer (10 mM Tris-CL, 0.5 mM EDTA, pH 9.0) samples were eluted. Shotgun metagenomics sequencing libraries were prepared by a PCR-free method: library composition and sequencing were conducted at Novogene (Cambridge, UK) using a HiSeq instrument (Illumina) with 150-bp paired-end reads and 6 G data per sample. To process shotgun metagenomics the MEDUSA pipeline was used.^35^ In short, the entire fecal genomic DNA was extracted from 100–150 mg of feces by repeated bead beating through modification of the IHMS DNA extraction protocol Q.^33^ Processing of raw reads was conducted with the NGless v.10 pipeline.^36^ Briefly, preprocessing of reads started by filtering basecalls with a *phred* score below 25 and also reads with length less than 45 bp. Subsequently, contaminants were removed by mapping the quality filtered reads against a database which contains plant, fungus, animal, human genomes (min match size = 45 and min identity = 95%). Filtered reads were mapped against the IGC (Integrated Gene catalog) with bwa.^37^ IGC contain 9.9 million human gut microbial genes.^38^ Gene abundance was computed with the NGless *dist1* option, multiple mapped reads were distributed based on the coverage of singly mapped reads. The gene abundance table which was generated with NGless was subsequently treated with MetaOminer V1.2^39^ for rarefaction to 10^7^ reads and RPKM normalization. A second Co-Abundance Gene groups (CAG) catalog was used together with IGC, those CAGs counting more than 500 genes were in this study considered as Metagenomic Species (MGS). Relative abundance of each MGS was calculated as the average abundance of the 50 most highly correlating genes. Classification of species for each MGS was prepared if at least 50% of the MGS also matched the same NCBI reference genome at 90% of length coverage and 95% identity. For the superior taxonomic levels, criteria were 75% and 85% of identity in order to assign phylum and genus respectively. Abundances of gut microbial derived metabolic modules (GMM) were determined by gene abundance tables based on the classification of Vieira-Silva.^40^

Computational protocols for the replication cohort were exactly the same as in the clinical study. General descriptions of the cohort have been previously published.^41^

### Epigenetic (methylation) arrays of PBMCs

DNA of PBMCs was isolated using QIAamp® DNA Mini Kit (250); QIAGEN, Ref# 51306, Lot# 169049798 according to instructions of manufacturer. DNA methylation was measured with the Infinium MethylationEPIC BeadChip (Illumina Inc., San Diego, CA) according to the manufacturers protocol. In short, 500 ng of genomic DNA was bisulfite converted using the EZ-96 DNA Methylation MagPrep Kit (Zymo Research, Irvine, CA, USA) with the KingFisher Flex robot (Thermo Fisher Scientific, Breda, Netherlands). The samples were plated in a randomized order. The bisulfite conversion was performed according to the manufacturers protocol with the following modifications. For binding of the DNA 15 µl MagBinding Beads was used. The conversion reagent incubation was done according to the following cycle protocol: 16 cycles of 95°C for 30 s followed by 50°C for 1 h. After the cycle protocol, the DNA is incubated for 10 min at 4°C. Next, DNA samples were hybridized on the Infinium MethylationEPIC v1.0 BeadChip (Illumina Inc., San Diego, CA) according to the manufacturers protocol with the modification that 8 µl bisulfite treated DNA was used as start material. Quality Control of the DNA methylation data was performed using ‘minfi’, ‘Meffil’ and ‘ewastools’ packages with R version 4.0.0. Age and sex were predicted based on the CpGs and compared to the actual data (Supplemental Methods Figure S3). Furthermore, for samples of the same individual, SNPs were analyzed (Supplemental Methods Figure S2). Samples who failed technical controls, including extension, hybridization and bisulfite conversion, according to the criteria set by Illumina, were excluded. Samples with a call rate <96% or at least with 4% of undetected probes were also excluded. Probes with a detection p-value >0.01 in at least 10% of the samples were set as undetected. Probes with a bead number <3 in at least 10% of the samples were excluded. Samples were normalized using the preprocess Noob function of minfi.^42^ Probes located on the sex chromosomes and CpGs with known SNPs (minor allele frequency >0.01) were omitted. Furthermore, gaphunter was applied to omit outlier probes and probes related to SNPs.^43^ B values were used for analyses.

### RNA isolation and real time quantitative polymerase chain reaction (RT-qPCR)

RNA was isolated following standard RNA isolation protocol. In short, PBMCs were lysed with 1 ml TriPure (Roche), after adding 0.2 ml chloroform to 1 ml Tripure solution, samples were centrifuged (15 min 12,000 × g, 4°C). The aqueous phase was transferred and mixed with 0.5 ml isopropanol and centrifuged (15 min 12,000 × g, 4°C). Afterwards the pellets were resuspended in 1 ml 70% ethanol and centrifuged (15 min, 7500 × g, 4°C). RNA was eluted in 20 µl RNAse free water. RNA concentrations were measured using the NanoDrop 1000 (Thermo Scientific). 1 μg of RNA was converted to cDNA with SensiFAST cDNA synthesis kit (Meridian Bioscience) according to the manufacturer’s instructions. qPCR was performed on a CFX Opus 384 PCR machine (BioRad) using SensiFAST SYBR No-ROX Green (Meridian Bioscience). *SLC7A5* gene expression was normalized with the expression of the housekeeping genes *RNA18S1* and *HPRT* according to the “deltadelta Ct” method and shown as fold-changes versus control (baseline). The expr-ession based on 18S or HPRT was averaged. Primers were manufactured by Sigma-Aldrich. Primers sequences used are: (F) TGACCTTGATTTATTTTGCATACC (R) CGAGCAAGACGTTCAGTCCT for *HPRT*, (F) GAGGGAGCCTGAGAAACGG (R) GTCGGGAGTGGGTAATTTGC for *RNA18S1*, (F) GCCTGTGTTCTTCATCCTGG (R) GTGGAGAAGATGCCCTGGAG for *SLC7A5.*

### Machine learning

Linear regression XGboost (v 0.90) models were used to predict the delta area under the curve (AUC) of mixed meal glucose levels with metagenomics pathways and microbes by implementation of gradient boosted trees. Nested cross-validation was performed to prevent overfitting, predictive models followed 100 iterations. For every iteration, data were split into a training (80%) and test (20%) set. Five-fold cross-validation in the training data was tested to improve and fit model hyperparameters, for model parameters, see supplemental methods. Explained variance was calculated as previously described.^44^ Two random variables were added to the predictive data, if random variables were predictive for the outcome, every feature with a lower feature importance than the random variable was regarded as irrelevant. Implementation of models was performed with Python (v 3.7.4) by scikit-learn (v 0.21.2) numpy (v 1.16.4) and pandas (v 0.25.1) libraries.

### Statistical analyses

Statistical analyses were performed in R (https://www.R-project.org/, v.4.2.1) using the libraries ‘nlme’ and ‘tidyverse’. For data visualization ‘ggplot2’ and ‘ggpubr’ were used. Mixed linear models were used throughout the study to assess changes in outcomes except if noted differently. Baseline differences between patients were considered random effects. FDR correction for multiple comparison correction was applied if necessary (e.g. for Spearman correlation heatmap of Figure 5 and S2). Percent time in range was defined as percent time over glucose 3.9 and under 10 mmol/l. Immunophenotyping of PBMCs was analyzed using paired t-test when comparing paired samples from baseline and 2 weeks post-histidine supplementation; when comparing differences between baseline and 2-week histidine treatment, antibiotics or recovery, Kruskal–Wallis one-way ANOVA was used as not all samples were paired (missing frozen PBMC vials).

## Results

### Plasma histidine levels increased after oral histidine supplementation

After the baseline mixed meal test, at visit 2 individuals started with the first histidine challenge of 4 g, after the initial intake, blood was drawn during 6 hours to follow histidine kinetics. After the initial dose, histidine intake was continued for seven weeks daily (Figure 1). Fasting plasma histidine levels are higher in T2D after two weeks, however, after correction for multiple testing this effect was lost (b = 20.90 uM, SE = 9.98, *p* = 0.04, q = 0.12). Other points were not significantly changed in both groups. Area under the curve for histidine during a 6-h curve was elevated after antibiotics in the control group (b = 16098947, SE = 2413738, *p* = 1.42 × 10^−8^) q = 4.25 × 10^−8^) and in the T2D group as well, as was the area under the curve after the recovery phase. Other time points did not show significant changes (Supplementary Table S1-S2).

### Glycemic control improved after two weeks of histidine supplementation

#### Glycemic control and body composition

**Figure 2.**
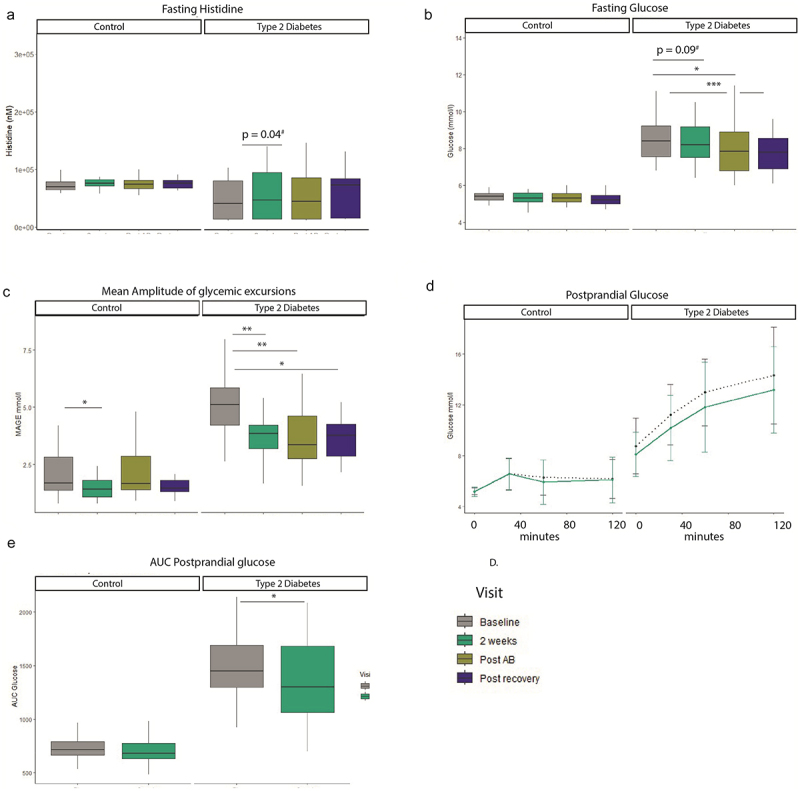
Histidine and clinical parameters. A. Fasting histidine increases after oral supplementation. B. Fasting glucose improves throughout the study in the type 2 diabetes group. C. Mean amplitude of glycemic excursion based on continuous glucose measurements improves in the type 2 diabetes group during the study. Results Mixed Meal test D. Glucose curve during 120 minutes mixed meal test. E. AUC glucose mixed meal test. Linear mixed models, ^#^nominal p value, */**FDR corrected. Control n = 19, Type 2 diabetes n = 20. *p < 0.05, **p < 0.01, ***p < 0.001. T2D: n = 20. Control n = 19.

As histidine supplementation has been shown to improve glycemic control, we measured different markers of glycemic control.^17^ In addition to this, parameters of metabolic control such as resting energy expenditure being measured by indirect calorimetry were measured. We found that resting energy expenditure did not differ between the visits in the T2D group or control group (Supplemental Table S3, Figure S4). Daily energy intake or macro nutrient intake such as protein, fat, fiber and carbohydrate intake did also not differ between the visits (Table S3). The total body fat content did not change during the study. We assessed parameters of glycemic control and found that after 2 weeks of histidine supplementation there was a trend in reduced fasting glucose in the T2D group (Figure 2, b = −0.50 mmol/l, SE = 0.29, *p* = 0.09, q = 0.11), subset analysis per ethnicity did not reveal new trends (Supplementary Table S1–3). Fasting glucose was decreased after antibiotics treatment and recovery phase in the T2D group compared to baseline. Similar results were observed for HbA1c, which was reduced after 2 weeks (b = −1.6 mmol/mol [−0.15%], SE = 0.75 mmol/l, *p* = 0.04, q = 0.05), post antibiotics and post recovery compared to baseline in participants with T2D. Insulin levels and HOMA-IR were not changed between visits. For a summary table of results (see Supplemental Table S4).

#### Continuous glucose measurement

Average glucose concentrations decreased during the course of the study in the T2D group but not in the control group. The average AUC per day increased after 2 weeks (b = −1234, SE = 567, *p* = 0.04, q = −.06), post antibiotics and post recovery. Percent time in range increased after 2 weeks (b = 9.2, SE = 4.4, *p* = 0.04, q = 0.06), post antibiotics and post recovery as well. Another parameter of glycemic control which is more focused on glucose amplitude excursions is the mean amplitude of glycemic excursions (MAGE, Figure 2). This parameter also improved after every visit: after 2 weeks (b = −1.3, SE = 0.39, *p* = 0.002, q = 0.003), post recovery and post recovery compared to baseline, see also Table S5.

### Postprandial glucose levels improve after oral histidine supplementation

To assess if improved parameters of glycemic control also relate to functional improvements of glycemic control we performed mixed meal tests, Figure 2. Here, we found that the postprandial AUC for glucose excursions during 2 h was decreased in the T2D group after 2 weeks of histidine supplementation (b = −132, SE = 53, *p* = 0.02), whereas no changes were seen in the control group (b = −23, SE = 23, *p* = 0.34). The incremental AUC was also decreased in the T2D group after 2 weeks (b = −132, SE = 53, *p* = 0.02, Figure S6). There were no changes in postprandial insulin AUC in T2D (b = −7406, SE = 4693, *p* = 0.13) and the control group (b = −1685, SE = 4123, *p* = 0.69).

### Broad immunophenotyping analysis of PBMC reveals that oral histidine supplementation specifically impact CD8+ MAIT cells

As monocytes play an important role in insulin resistance development,^45^ we set out to address if histidine supplementation improves inflammatory markers as previously shown.^17^ We found that blood leukocyte count showed a trend toward decreased numbers after 2 weeks (b = −0.5, SE = 0.26, *p* = 0.05, q = 0.10) and after the recovery phase (b = −0.75, SE = 0.26, *p* = 0.006, q = 0.02) in the T2D group, while no changes were observed in the control group (Table S4). To better understand if important drivers of inflammation such as monocytes were affected, we assessed monocyte markers by flow cytometry. The percentage of CD11b+ monocytes, CD16+ and CD16-negative monocytes did not change in the T2D group after histidine intake, however in healthy controls the percentage of CD16+ monocytes increased while the proportion of CD16- monocytes was decreased by histidine supplementation (Figure S7). In addition to this, the percentage of CD8+ T cells, CD4+ T cells and CD4 CD8 double-positive/double-negative T cells did also not change after 2 weeks of histidine supplementation (Figure S8). Similarly, the expression of the activation markers CD69 and CD25 was not altered in CD4 nor CD8 T cells (Figure S8).

**Figure 3.**
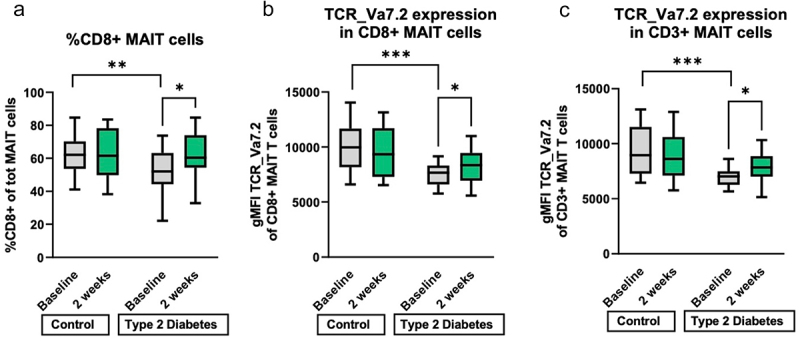
Immunophenotyping PBMC. A. Percentage of CD8+ MAIT cells is at baseline higher in the control group and increases during two weeks of histidine in the type 2 diabetes group. B. the expression (geometric mean fluorescence intensity) of the MAIT cell specific T cell receptor (TCR Va7.2) is higher at baseline in the control group than in the T2D group in CD8+ MAIT cells. After two weeks of histidine supplementation is the T cell expression in the T2D group also increased compared to baseline. C. the expression (geometric mean fluorescence intensity) of TCR Va7.2 on total MAIT T cells is decreased in T2D at baseline and increased after oral histidine in the T2D group. T2D: type 2 diabetes. T2D: n = 20. Control n = 19. *p < 0.05, **p < 0.01, ***p < 0.001. Paired non-parametric t test. MAIT: mucosa associated invariant T cell. PBMC: peripheral blood monocyte cells. gMFI: geometric mean fluorescent intensity. TCR: T cell receptor.

As MAIT cells have been shown to be affected in metabolic diseases such as T2D^20^ and activation is dependent on the amino acid transporter SLC7A5 which has a high affinity of histidine,^46^ we measured the proportion of circulating MAIT cells, which were identified by simultaneous expression of CD161 and TCR Va7.2. Within the T cell population, the percentage of CD161+ TCR Va7.2+ (total) MAIT cells as well as the proportion of CD8+ within MAIT cells was higher in healthy controls than in T2D individuals at baseline (Figure 3(a), Figure S9). Moreover, histidine supplementation increased the proportion of CD8+ MAIT cells to the levels of healthy subjects (Figure 3(b), Figure S9). The expansion of CD8+ MAIT cells was maintained after 1 week of antibiotic treatment and declined after 2 weeks (albeit not significantly) (Figure S9). In addition, the surface expression of the invariant TCR chain Vα7.2 on single CD8+ MAIT cells was increased after two weeks of histidine in the T2D group visits (mean diff = 9.1, SEM = 4.6, *p* = 0.048), as shown by the mean fluorescence intensity (MFI) of TCR Va7.2 staining (Figure 3(c)). The same effect on TCR Va7.2 was found in the total MAIT cell population (Figure 3(c)).

### Bacteria with flavin biosynthesis potential are associated with CD8+ MAIT cells

**Figure 4.**
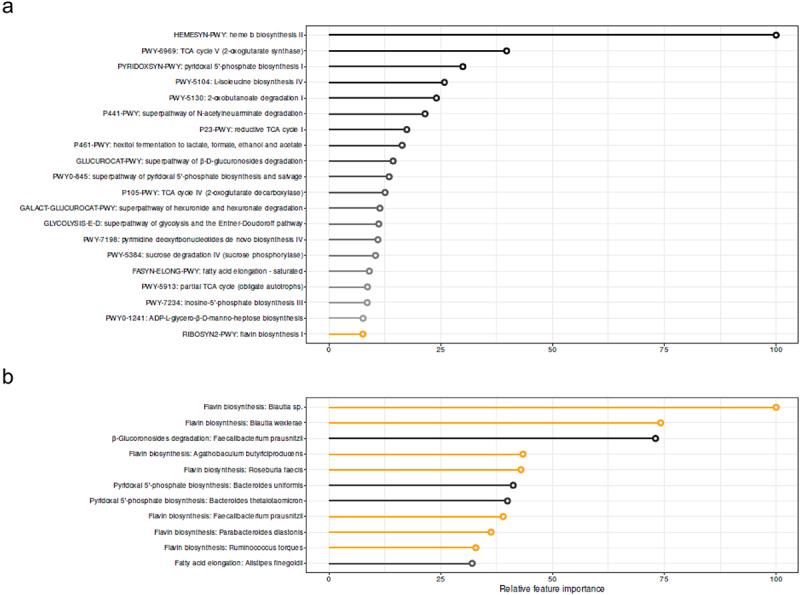
Discriminative features for CD8+ MAIT percentages based on A. delta Fecal metagenomics pathways with explained variance: 25.7% B. Delta fecal metagenomics of bacteria belonging to the 20 pathways depicted in A. explained variance 14.6%. XGBoost regression models were applied. Black circumference indicated relation to flavin biosynthesis pathway.

As MAIT cells are located in the gut and bacterial metabolites can bind to the TCR of MAIT cells,^27,28^ we hypothesized that gut microbiota influence circulating MAIT cell numbers. To evaluate if changes induced by oral histidine influence the relationship between gut microbiota and MAIT cells, we used the delta of pathway abundance and CD8+ MAIT cells from the baseline visit and 2 weeks. We found that the explained variance to predict mean MAIT CD8+ delta was 25.7%. Interestingly, the heme b biosynthesis pathway was most predictive for CD8+ MAIT cells. Other discriminative pathways included pyridoxal 5’-phosphate, pyrimidine deoxyribonucleotides and flavin biosynthesis among others (Figure 4).

When using bacteria belonging to the 20 most predictive microbial pathways for CD8+ MAIT cells, the explained variance of CD8+ MAIT cells was 14.6%. The threshold for valuable discriminative microbes was reached at position 10 of the most discriminative microbes by finding of a random variable at position 12. Within the first 11 discriminative microbes, six were related to the flavin biosynthesis pathway (Figure 4). The abundance of individual bacteria was not significantly different between baseline and two-week histidine treatment, although a trend toward increased levels of *Bacteroides thetaiotaomicron* was observed (*p* = 0.09); thus underscoring that metabolic functions rather than single bacteria abundance are linked to changes in MAIT cells.

**Figure 5.**
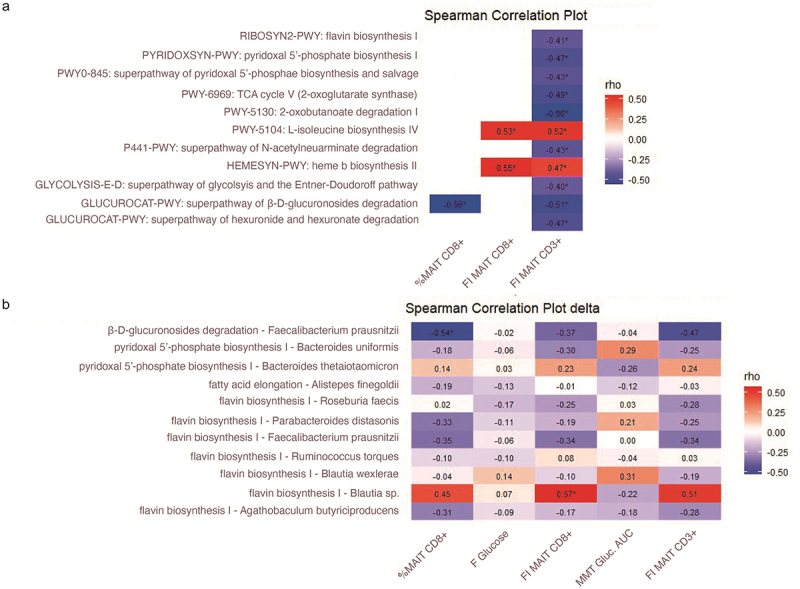
Correlation heatmap between A. Pathways and MAIT cell proportions and markers B. Bacterial species and genera belonging to the most discriminative pathways and MAIT cell markers. *FDR p < 0.05. Spearman correlation. Pathways and bacterial species and genera were based on fecal metagenomics analysis. FI: mean fluorescence intensity of TCR Va7.2. AUC: area under the curve. MMT: mix meal test.

Eleven of the 20 most discriminative features of the machine learning algorithm were associated with mean fluorescent intensity of TCR Va7.2 in the MAIT cell population, after FDR correction. Five were also associated with the percentage of CD8+ MAIT cells (Table S6). Species *Faecalibacterium prausnitzii* and *Blautia spp*. were significantly associated with changes in CD8+ MAIT cell. *Faecalibacterium* prausnitzii were inversely associated with the percentage of CD8 MAIT cells, while *Blautia spp* were positively associated with the expression rate of TCR Va7.2 in the MAIT cells (Figure 5, Table S7).

### Gut microbiota pathway and MAIT cell association in an external replication cohort

As gut microbiota can be diverse and vary between groups,^47^ we sought to replicate our findings in a larger external cohort. For this, we used cross-sectional MetaCardis cohort^20,48^ in which fecal metagenomic data and flow cytometry measurements of MAIT cells were available in 162 individuals. Within this group, 113 individuals had T2D and 49 were healthy controls. In the MetaCardis cohort, nine pathways and three bacterial species, which were predictive for MAIT cells in our clinical trial, were associated to MAIT cells. The flavin biosynthesis pathway was also associated with %CD3+ MAIT cells (Figure S12). The association included inverse correlations between CD3+ MAIT cells and riboflavin biosynthesis, pyridoxal 5’ phosphate biosynthesis and L-isoleucine biosynthesis (Figure S6). In addition to this, the relative abundance of *Faecalibacterium prausnitzii*, which showed a strong inverse association with the subset of CD8+ MAIT cells in our study, had a strong inverse correlation with the percentage of activated CD25+ MAIT cells in the MetaCardis cohort. In addition, *F. prausnitzii* was associated to the proportion of MAIT cells in the MetaCardis cohort (Figure S12).

### Epigenetic (DNA methylation) of PBMCs was affected by oral histidine intake and related to CD8+ MAIT cells as well as postprandial glucose AUC

**Figure 6.**
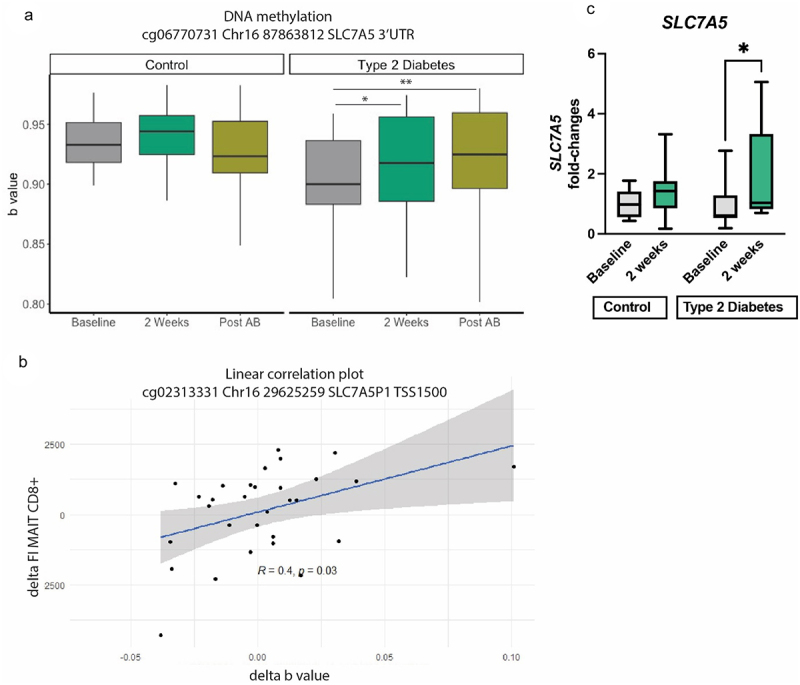
SLC7A5 methylation of A. a specific CpG: cg06770731 Chr16 87,863,812 SLC7A5 3’UTR Linear mixed models *p < 0.05, **p < 0.01, ***p < 0.001. Post AB: post antibiotics. B. Spearman correlation between a CpG and mean fluorescent intensity (FI) of CD8+ MAIT cells. Delta of both variables was used for this analysis. C. Gene expression of SLC7A5 in healthy controls and T2D individuals at baseline and after 2 weeks of histidine supplementation. Significant increase in SLC7A5 expression in the T2G group assessed with Mann–Whitney test; *p < 0.05.

As activation of MAIT cells is dependent on nutrient intake via SLC7A5^46^, an amino acid transporter using histidine as the preferred substrate^49^ and of which expression is epigenetically controlled,^50^ we hypothesized that DNA methylation of SLC7A5 is affected by higher histidine availability. To assess this, we studied DNA methylation of PBMCs collected at baseline, after 2 weeks of histidine supplementation and after antibiotics. We selected cytosine nucleotide guanine (CpG) islands associated with SLC7A5. After quality control, 89 CpGs were identified related to SLC7A5. Using the delta between baseline and after 2 weeks, we that found eight CpGs were associated to the rate of TCR Va7.2 expression by CD8+ MAIT cells (Figure 6(a,b), Table S8), and three CpGs associated to postprandial glucose AUC. Of the CpGs analyzed, fifteen CpGs were in the promotor region, whereas 74 were in body, untranslated region or exon. To investigate whether the higher SLC7A5 methylation rate translated to changes in gene expression following histidine supplementation, we performed QPCR analysis and found that in the T2D group the expression of *SLC7A5* was significantly increased upon histidine intake, while it remained unchanged in the control group in line with the unchanged SLC7A5 methylation in healthy controls (Figure 6(c)).

## Discussion

**Figure 7.**
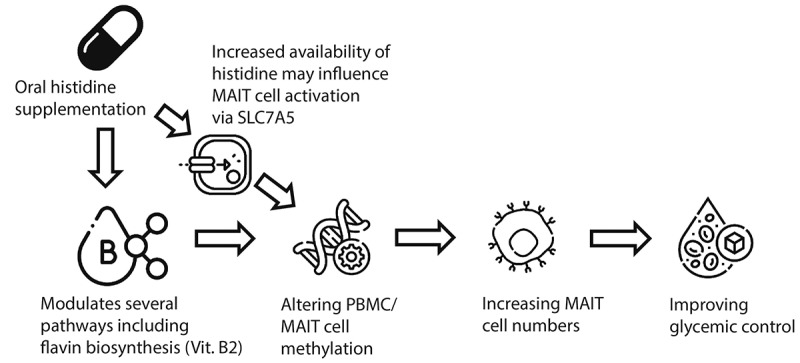
Proposed framework how oral histidine may influence glycemic control. Oral histidine may modulate gut microbiota pathways including vitamin B2 which can bind to MAIT cells, altering MAIT cell methylation. Additionally, abundant systemic availability of histidine may influence MAIT cell activation via SLC7A5 expression, thereby affecting SLC7A5 gene methylation. Altered MAIT cell methylation facilitates proliferation of MAIT cells which is generally associated with metabolic health and could thus thereby improve glycemic control.

In the present study, we provide evidence about the impact of oral histidine on gut microbiota, MAIT cells and glycemic control. We show that upon oral histidine intake fasting plasma histidine concentrations increase. Histidine supplementation subsequently exerts several beneficial effects on glycemic control in participants with T2D with reductions of fasting glucose, HbA1c, time in range and the mean amplitude of glycemic excursions. In addition to this, postprandial mixed meal glucose concentrations were lower in the T2D group after two weeks while insulin levels were not affected, suggesting increased insulin sensitivity in the T2D group while no change was observed in healthy controls. Changes in postprandial glucose excursions occurred within two weeks along with the significant increase in MAIT cell numbers, which was inversely associated with HbA1c and serum glucose levels.^20^ MAIT cells reside in the gut and thus MAIT cells could be directly affected by gut microbiota changes induced by histidine.^51^ We hypothesized that histidine affects metabolic pathways of gut microbes and improves glycemic control via MAIT cells. Interestingly, we found an association between MAIT cells and several gut microbiota related pathways such as flavin (vitamin B2) and pyridoxal phosphate (vitamin B6) biosynthesis, which was replicated in an external independent cohort. This is of interest, as previous research has shown that intermediates of the riboflavin biosynthesis pathway can activate MAIT cells.^27^ In addition to this, several methylation sites of the SLC7A5 gene were differentially methylated, suggesting that histidine supplementation directly or indirectly affects this amino acid transporter in PBMCs. Figure 7 provides a conceptual framework on how oral histidine aids glycemic control by affecting the metabolic capacity of the gut microbiota, epigenetics and expansion of circulating MAIT cell.

Metabolites from the riboflavin biosynthesis pathway are necessary for MAIT cell intra-thymic development and subsequent peripheral expansion in mice.^25,52^ Indeed, colonization of riboflavin synthesizing bacteria such as *Escherichia coli* or *Proteus mirabilis* promotes MAIT cell development.^25,52^ In our study we observed an increase of CD8+ MAIT cells after two weeks of histidine supplementation in participants with T2D. This increase was inversely associated to riboflavin biosynthesis, suggesting that lower riboflavin bioavailability in the gut is associated to higher number of MAIT cells in the circulation. As MAIT cells recognize bacterial riboflavin biosynthesis precursors, we may speculate that decreased riboflavin synthesis results in less MAIT cell activation and production of inflammatory cytokines by MAIT cells. This is in line with findings in the literature, as a reduced availability of riboflavin decreases MAIT cell activation.^53^ Moreover, we found that *F. prausnitzii* was inversely associated with MAIT cells and inversely associated with the expression of the activation marker CD25 in MAIT cells in the MetaCardis cohort. The negative association between *F. prausnitzii* and CD8 MATI cells may appear contradictory as *F. prausnitzii* is a butyrate producer and generally associated with metabolic health.^54^ Nonetheless, an increased activation of T cells will contribute to a higher inflammatory tone, which is generally detrimental for metabolic health,^55^ hence the inverse association with CD25 expression in MAIT cells may underscore a beneficial effect of *F. prausnitzii.*

MAIT cells have shown to be associated with cardiometabolic metabolism; in a cross-sectional study low MAIT cell abundance was associated with increased HbA1c, cardiac function and inflammatory markers.^20^ In addition to this, obese individuals and individuals with T2D have lower circulating MAIT cells which show defective activation patterns compared to MAIT cells of non-T2D and non-obese individuals.^22^ Apoptosis of MAIT cells is dose dependently induced by increasing glucose concentration in vitro.^20^ In line, in our study we found increased numbers of MAIT cells and improved glycemic control. Based on previous literature which found low circulating MAIT cell rates to be associated to deteriorated glycemic control^45^ and increased inflammatory markers,^20^ a rise in MAIT cell proportions could lead to improved glycemic control by dampening low-grade inflammation, which is a common trait of metabolic disorders, such as T2D. This reduction in inflammatory tone could contribute to the improved glycemic control via reduced activation of MAIT cells as a consequence of a decreased flavin biosynthesis and/or SLC7A5 expression (Figure 7). In regard to the latter, we found that histidine intake resulted in changes in SLC7A5 gene methylation within PBMCs, resulting in increased SLC7A5 gene expression in the T2D group. As we did not find any effects of histidine on the proportion nor activation of CD4 and CD8 T cells, but solely on MAIT cells, we hypothesize that the changes in SLC7A5 methylation occurred mainly in MAIT cells.

Postprandial glucose levels were lower after two weeks of histidine supplementation in our study. However, insulin levels or HOMA-IR were not affected. This indicates that glycemic control was improved independent of the pancreas, excluding the pancreas as target of action for histidine. Insulin sensitivity could be increased by histidine as second messenger or histidine could increase the demand of glucose by stimulation of anabolic processes.^6^ However, we did not find any differences in resting energy expenditure. Therefore, as MAIT cells are associated to glycemic control and affected by T2D,^45,56^ this link appears more direct. However, the direct link between glycemic control and MAIT cells still remains to be determined.

Nutrient uptake for T cells is paramount for activation and adequate immune regulation, as T cells without the amino acid transporter SLC7A5 are unable to differentiate and activate mTORC1.^57^ Moreover, MAIT cell glycolysis is essential for MAIT cell activation and glycolysis mediated MAIT cells activation is in turn dependent on mTORC activation.^58^ Interestingly, amino acid signaling plays an important role in MAIT cell activation, as mTORC activation in MAIT cells is dependent on the amino acid transporter SLC7A5^58^ also known as L type amino acid transporters 1 (LAT1). SLC7A5 plays an important role in cell growth and development by absorption of eight out of nine essential amino acids.^49^ In fact histidine is the preferred substrate of SLC7A5^49^ and expression of SLC7A5 gene expression is regulated by DNA methylation in the promotor region.^50^ In addition, hyperglycemia reduces SLC7A5 expression,^59,60^ which may contribute to sarcopenia in diabetes. We found several CpGs to be differentially methylated after histidine supplementation. As SLC7A5 has a high affinity with histidine and is necessary for MAIT cell activation^49,58^ and we found histidine levels to be elevated in our study, a direct effect of increased amino acid availability on MAIT cell function is also conceivable opposed to the effect via gut microbiota. This could result in increased proliferation and higher circulating MAIT cell numbers, as observed in our study (Figure 7). However, SLC7A5 may also be affected by glucose levels itself.^60^ In addition to this, hypermethylation of the 3’ untranslated region (UTR) is associated with overexpression of genes,^61^ therefore the observed hypermethylation in SLC7A5 CpGs may be driving the significantly higher *SLC7A5* gene expression upon histidine intake in the diabetic participants. This could be a response to the increase of systemic histidine availability. As amino acids are necessary for MAIT proliferation,^46^ increased MAIT cell numbers could be facilitated by hypermethylation SLC7A5 related CpGs. Interestingly, not all CpG’s with hypermethylation were associated with MAIT cell numbers, *cg06770731* was for example not associated to MAIT cell numbers whereas *cg02313331* was associated to MAIT cell numbers and postprandial glucose AUC. However, *cg02313331* was not differentially hypermethylated between visits, suggesting that not all CpGs are uniformly associated to MAIT cells and glycemic control. Additionally, both the increase in circulating CD8 MAIT cells and methylation appeared greater after oral histidine with little effect of the one-week antibiotic treatment, this may be explained by the fact that methylation are lasting epigenetic modifications and the higher expression of SLC7A5 may aid MAIT cell proliferation and maintenance of MAIT pool size. While the effects on CD8 MAIT cell expansion in T2D are likely microbiota-dependent, given the observed association with microbial riboflavin biosynthesis pathway, it is likely that the increased rates in SLC7A5 gene methylation are mediated solely by increased circulating histidine; although we can also not exclude a microbial indirect effect.

In our study, we found the association between pathways to be stronger related to MAIT cells than species alone. As bacteria rely on cross-feeding and are limited to certain substrates which bacteria can metabolize, the upregulation of an entire metabolic pathways such as flavin biosynthesis is more likely to be consistently associated to an outcome such as the abundance of MAIT cells than a more varying single species. Furthermore, the pyrimidine biosynthesis pathway was among the discriminative features for MAIT cell abundance. Given that histidine itself is the strongest inhibitor of histidine synthesis in bacteria^62^ and substrates among several bacterial biosynthesis pathways such as L-histidine, riboflavin purine and pyrimidine are interchangeable,^63^ it is likely that histidine supplementation stimulates production of other pathways as more substrate for metabolizationflast become available.

After antibiotics and after the recovery phase histidine levels were not significantly reduced which is in contrast to the decrease observed after 2 weeks. This suggests a decline in systemic histidine levels. As most participants had minor gastrointestinal complaints such as thinner stool, transit time is likely to be increased which decreases the absorption of histidine. Interestingly, the improvement of fasting glucose or MAGE after antibiotics and the recovery phase suggests that the improved effect continues after two weeks. This could be due to a broader effect on physiology of antibiotics, as less nutrients may be absorbed. However, relevant metabolic parameters such as resting energy expenditure and total body fat content did not change during the study, suggesting that metabolism was not majorly affected by antibiotics. Additionally, before the start of the study we expected to reverse a potential effect by application of antibiotics in week 2 if the effect is dependent on gut microbiota. In contrast, the improvements in glycemic control are not abolished after antibiotics application, which may be due to the reason that bacteria which mediate the effect of histidine are resistant to antibiotics application. Alternatively, the initiated effect of histidine on glycemic control which started in the first two weeks may continue during the seven days of antibiotics application. Future studies should focus on replicating the proposed mechanism in this study.

ImP has been shown to be detrimental for insulin signaling if injected in mice,^19^ however, direct supplementation of histidine improved glycemic control in our studies and also in women with MetSyn.^17^ The circumstances under which ImP is produced such as the local environment in the colon may determine if the effect of ImP is metabolically advantageous or detrimental. Alternatively, histidine could have direct beneficial effects on glycemic control that outweigh the detrimental effects of imidazole propionate.

### Limitations

This study was a parallel intervention study but did not have a randomized and blinded placebo group. This limits the generalizability of findings as it cannot be excluded that the observed effects are influenced by a participation in the trial alone. However, this study was not primarily designed to assess clinical outcomes which may introduce bias. In addition to this, the duration of 2 weeks for the initial period was relatively short compared to an intervention trial performed by Feng et al.^17^ with oral histidine in women with MetSyn. Participants in the study of Feng et al. used a daily dose of 4 g histidine for 12 weeks. However, the observed improvement of glycemic control was in HOMA-IR and not in fasting glucose.^17^ Another factor that could influence results is that all participants in the T2D group are on statin and metformin treatment, two drugs that affect gut microbiota composition.^64^ Metformin is among the first treatment lines for glycemic control in T2D and patients with T2D frequently require statin therapy,^65^ therefore the microbiome structure under the influence of these two drugs may be a more representative microbiota composition of most diabetes patients.

This study shows that oral histidine supplementation improves glycemic control and changes in gut microbiota composition associated with MAIT cell number increase, which was replicated in an external cohort. Changes in MAIT cell number may be facilitated by epigenetic changes due to altered amino acid availability. To discern between microbiota related effects and direct effects of histidine on MAIT cells, future studies should focus on modulation of riboflavin availability in the gut and the effect of histidine availability for MAIT cell activation. Harnessing the beneficial effects of histidine may contribute to improved glycemic control and gut microbiota composition in T2D by administration of advanced food supplements or personalized nutrition advices thus moving closer toward a personalized nutrition approach in T2D.

## Data Availability

The data that support the findings of this study are available on request from the corresponding author. The data are not publicly available due to restrictions e.g. their containing information that could compromise the privacy of research participants.
